# Congenital Anomalies of the Kidney and Urinary Tract: A Genetic Disorder?

**DOI:** 10.1155/2012/909083

**Published:** 2012-05-20

**Authors:** Ihor V. Yosypiv

**Affiliations:** Section of Pediatric Nephrology, Department of Pediatrics, Hypertension and Renal Center of Excellence, Tulane University Health Sciences Center, New Orleans, LA 70112, USA

## Abstract

Congenital anomalies of the kidney and urinary tract (CAKUTs) occur in 3–6 per 1000 live births, account for the most cases of pediatric end-stage kidney disease (ESKD), and predispose an individual to hypertension and cardiovascular disease throughout life. Although CAKUTs are a part of many known syndromes, only few single-candidate causative genes have been implicated so far in nonsyndromic cases of human CAKUT. Evidence from mouse models supports the hypothesis that non-syndromic human CAKUT may be caused by single-gene defects. Because increasing numbers of children with CAKUT are surviving to adulthood, better understanding of the molecular pathogenesis of CAKUT, development of new strategies aiming at prevention of CAKUT, preservation of renal function, and avoidance of associated cardiovascular morbidity are needed. In this paper, we will focus on the knowledge derived from the study of syndromic and non-syndromic forms of CAKUT in humans and mouse mutants to discuss the role of genetic, epigenetic, and *in utero* environmental factors in the pathogenesis of non-syndromic forms of CAKUT in children with particular emphasis on the genetic contributions to CAKUT.

## 1. Introduction

Congenital anomalies of the kidney and urinary tract (CAKUTs) occur in 3–6 per 1000 live births and are responsible for 34–59% of chronic kidney disease (CKD) and for 31% of all cases of end-stage kidney disease (ESKD) in children in the United States (Table 1) [1–8]. All children with ESKD require renal replacement therapy and up to 70% of them develop hypertension [9]. Given that the survival rate of children with ESKD is about 30 times lower than that of healthy children [10], new strategies are needed to prevent CAKUT, preserve renal function, and reduce associated cardiovascular morbidity.

CAKUTs comprise a wide range of renal system structural and functional malformations that occur at the level of the kidney (e.g., hypoplasia and dysplasia), collecting system (e.g., hydronephrosis and megaureter), bladder (e.g., ureterocele and vesicoureteral reflux), or urethra (e.g., posterior urethral valves) [16]. With improved prenatal screening, many cases of CAKUT are diagnosed by antenatal ultrasonography performed on 18–20 weeks of gestation. Most common antenatal manifestations of CAKUT include oligohydramnios or variations in gross morphology of the kidney, ureter, or bladder. Postnatal manifestations of CAKUT may include presence of palpable abdominal mass or single umbilical artery, feeding difficulties, decreased urine output, deficient abdominal wall musculature, and undescended testes in a male infant or multiorgan birth defects [17]. Despite the broad spectrum, all forms of CAKUT result from faulty renal system development [16, 18, 19]. Although many forms of CAKUT occur in the context of multiorgan malformation syndrome (http://www.ncbi.nlm.nih.gov/omim), most cases of CAKUT are nonsyndromic [16]. Syndromic CAKUTs develop in association with additional congenital abnormalities outside of the kidney and urinary tract and manifest clinically recognizable features of a known syndrome, whereas in nonsyndromic CAKUT congenital structural anomalies are confined only to the kidney and urinary tract. Although only few single-candidate causative genes have been implicated so far in nonsyndromic cases of human CAKUT [20, 21], evidence from mouse models supports the hypothesis that nonsyndromic human CAKUT may be caused by single-gene defects.

## 2. Evidence from Mouse Models to Suggest Monogenic Causes of CAKUT

Genetic manipulations in mice identified a number of genes and gene networks that orchestrate normal development of the kidney and urinary tract (renal developmental genes (RDGs)) and provided new insights into the pathogenesis of CAKUT ([22, 23], http://www.gudmap.org/). Because concerted inductive interactions of many RDGs expressed in the mesenchyme (anlagen of the nephron), stroma (anlagen of the renal interstitium), ureteric bud (UB, anlagen of the renal pelvis, calyces, ureter, and collecting ducts), and cloaca (anlagen of the bladder and urethra) at multiple developmental stages are required for normal morphogenesis of the kidney and lower urinary tract [22], single-RDG mutations might affect kidney development at multiple steps and cause a broad phenotypic spectrum of CAKUT that ranges from vesicoureteral reflux (VUR) to renal agenesis [16]. For example, mice with global genetic deletion of *Ret,* a receptor tyrosine (Tyr) kinase (RTK) for glial-derived neurotrophic factor (*GDNF*) produced in the mesenchyme, expressed in the UB exhibit a spectrum of anomalies ranging in severity from uni- or bilateral renal and ureteral agenesis, to blind-ending ureters with no kidney tissue, to hypoplastic/dysplastic kidney rudiments [24]. Moreover, specific CAKUT phenotype in *Ret* mutants depends on distinct Ret-stimulated signaling pathways. ^Tyr1062^ *Ret* mouse mutants, characterized by aberrant phosphatidylinositol 3-kinase (PI3K)/Akt and rat sarcoma (Ras)/extracellular-signal-regulated kinase (Erk) 1/2 signaling, exhibit more severe defects, which include renal agenesis, hypodysplasia, and ureteral defects, whereas ^Tyr1015^ *Ret* mutans, characterized by aberrant phospholipase C (PLC) *γ* signaling, manifest renal hypodysplasia and ureteral defects, but not renal agenesis [25]. The diverse phenotypes of these *Ret* mutants resemble human CAKUT, including incomplete penetrance (lack of disease manifestation in the presence of gene mutation) and variable expressivity (variation in type and severity of disease between individuals with the same gene mutation). Despite new insights into the molecular basis of CAKUT obtained in model organisms, integrated understanding of the role of genetic factors in the pathogenesis of nonsyndromic forms of CAKUT in humans is far from complete.

## 3. What Is the Evidence That Nonsyndromic Human CAKUTs Have a Genetic Basis?

The possibility of genetic basis of nonsyndromic CAKUT is supported by occurrence of familial cases of nonsyndromic renal agenesis, hypodysplasia, renal tubular dysgenesis, multicystic dysplastic kidney (MCDK), or VUR [26–30]. The observations that diverse forms of CAKUT occur in the same family [31] suggest that specific genetic mutations can potentially lead to CAKUT, but the final renal system phenotype depends on either genetic background or environmental factors. Despite recent identification of mutations in a number of genes in patients with nonsyndromic forms of CAKUT (Table 2), evidence to suggest that all cases of nonsyndromic CAKUT in humans are due to single-gene mutations is missing. Most studies report presence of known potential CAKUT-causing RDG mutations only in minority (1.9–20%) of patients with nonsyndromic CAKUT. Mutations in *HNF1*β*, Pax2, UMOD, or Eya1 *are detected in 1.9% of 538 patients from 456 families of predominantly European population with nonsyndromic CAKUT [32]. Mutations in hepatocyte nuclear factor-1 *β* (*HNF1*β**) are identified in 10% of Japanese children with renal hypodysplasia and unilateral MCDK [33]. Mutations in *HNF1*β**, *Eya1*, *Six1*, *Sall1* and *Pax2* are identified in 5–15% of children from European population with nonsyndromic CAKUT [20]. Massively parallel exon sequencing of 30 candidate genes in pooled DNA from children with unilateral renal agenesis, renal hypodysplasia, or VUR in the United States identified novel mutations in 4 genes (*Ret*, *BMP4*, *FRAS1,* and *FREM2*) in 17% of cases [21]. *Pax-2*, but not its downstream target gene, *GDNF*, polymorphism (a variation in the DNA sequence at a given locus that is too common to be due merely to new mutation) is associated with reduced kidney size in neonates [34, 35]. The differential effects of *Pax2* and *GDNF* polymorphism on CAKUT phenotype may be due, in part, to unidentified cell-specific cofactors that regulate gene expression. Of interest, polymorphism in GDNF receptor *Ret* is associated with reduced kidney size in neonates [36]. These findings may be interpreted to suggest that while RTKs other than Ret are unable to rescue renal phenotype in the absence of Ret, growth factors other than GDNF can act *via* Ret to do so. Notably, mutations in *Ret* are found in 35% of humans with various forms of renal agenesis [37]. Association of single polymorphism in the human *AT_2_R* gene, 1332A > G transition, with CAKUT has been reported in children with ureteropelvic junction (UPJ) stenosis, megaureter, MCDK, renal agenesis, and hydronephrosis from Germany, Italy, Korea, and the United States [38–41]. In contrast, several studies were unable to detect any known mutations in several RDGs linked to CAKUT in mice in humans. No known *UMOD* (European population) or *AT_2_R* (Japanese population) gene mutations were identified in children with diverse forms of nonsyndromic CAKUT [42, 43]. There was no association of primary VUR with mutations in *Pax2*, *HNF1*β*, Ret,* or *Robo2* in children from European population [44]. Thus, the contribution of genetic mutations to the cause of nonsyndromic CAKUT in the majority of children remains unresolved.

Several studies report a discrepancy in the impact of genetic mutations on CAKUT phenotype between mice and humans. For example, despite severe renal phenotype observed in *Robo2/Slit2-*mutant mice, which includes formation of supernumerary ureters [59], these gene mutations are very rarely associated with familial nonsyndromic VUR in children [60]. Mutations in the genes encoding for angiotensinogen (AGT), renin, ACE, or angiotensin II receptor type 1 (AGTR1) in mice result in severe medullary hypoplasia and hydronephrosis [68], a phenotype not observed in humans with *AGT*, *renin*, *ACE,* or *AGTR1* mutations [29]. The reasons for different CAKUT phenotypes observed in mice and humans with mutations in the same RDG may include severity of mutation (e.g., loss-of function mutation in mice models and missense mutations in humans), a higher complexity of epistatic or epigenetic interactions in humans compared to mice, or other factors. Importantly, the discrepancy between the mice and human CAKUT phenotype calls for caution when extrapolating findings observed in mice to humans.

## 4. Mechanisms That Dictate the Phenotypic Spectrum of CAKUT

Phenotypic heterogeneity of CAKUT can result from the following mechanisms: mutations in a single or multiple genes linked to human CAKUT [16], genetic [69–74] or epigenetic modifiers [75], mode of inheritance and environment [76].

### 4.1. Mutations in a Single or Multiple Genes Linked to Human CAKUT

#### 4.1.1. Locus Heterogeneity

 Although such hereditary cystic kidney diseases as ADPKD and nephronophthisis are not a part of CAKUT phenotype spectrum and should not be confused with CAKUT, important lessons can be drawn from our current knowledge of these and other forms of hereditary renal disease. In this regard, genetic locus heterogeneity (mutations in genes at different chromosomal loci) is a major determinant of interfamilial disease variability in ADPKD, accounting for earlier onset of ESKD in patients with ADPKD1 compared with patients with ADPKD2 [77] and of disease severity in children with nephronophthisis-related ciliopathies [78]. Whether genetic locus heterogeneity plays a role in interfamilial variability in CAKUT remains to be determined.

#### 4.1.2. Allelic Heterogeneity

 The specific combination of mutations dictates phenotypic outcome in some forms of CAKUT. For example, the presence of two truncating mutations in *PKHD1* results in nonfunctional fibrocystin and leads to death in the neonatal period [77, 79]. In contrast, patients with two missense (hypomorphic alleles that produce partially functional fibrocystin) mutations or a missense and a truncating mutation have a more favorable prognosis. Histologically, the severity of collecting duct dilatation and of degenerative changes in cortical tubules is more pronounced in neonates with truncating than missense *PKHD1* mutations [79]. Unlike in ARPKD, no clear correlation between mutation type and the severity of kidney disease is detected in ADPKD1 or 2 [77, 80]. Considerable disease variability in patients with the same *PKD1* or *PKD2* mutations supports the notion that additional genetic and environmental factors may modulate phenotypic outcome in ADPKD. Given that two null mutations in only *NPHP6*, but not in *NPHP2*-*NPHP5*, caused a more severe renal phenotype compared to null/missense mutations, the authors proposed that genetic locus heterogeneity is the major determinant of the disease phenotype with allelic heterogeneity being important only for certain genes [78].

#### 4.1.3. Allelic Variation

 Allelic variation in gene expression (significant difference in gene expression between the two alleles, which is transmitted by Mendelian inheritance) is common in the human genome [81]. Thus, allelic variation may modulate the level of various CAKUT mutants, leading to broad phenotypic spectrum of CAKUT.

### 4.2. Genetic Modifiers

Modifier genes can potentially modulate the CAKUT phenotype despite a unique CAKUT genotype. In this case, mutation in one gene will cause CAKUT or alter the phenotype only in the presence of genetic change in another gene (epistatic gene interactions). One of the well-recognized examples involves worsening the severity of ADPKD in contiguous deletions of *PKD1* and adjacent tuberous sclerosis gene, *Tsc2* [82]. Interactions between mouse orthologs of the genes linked to human CAKUT such as* PKHD1* and *HNF1*β*, PKD1* and *PKD2 *or polycystin 1, the product of the* PKD1* gene and tuberin, the product of the* Tsc2* gene have been reported in animal models [72–74, 83]. Large intrafamilial variability in renal disease progression in siblings with ADPKD, coupled with a significant excess of variability in siblings compared with monozygotic twins, provides further support for a role of genetic modifiers in children with ADPKD [84]. The fact that the spectrum of CAKUT phenotypes associated with *HNF1*β** or uromodulin (*UMOD*) mutations and age of their manifestations differ [33, 55, 85, 86] may be due, in part, to the ability of *HNF1*β*, a *developmentally regulated transcription factor, to regulate expression of *UMOD* or aggravate the phenotype of ADPKD [87, 88]. Available evidence suggests that epistatic gene interactions may be important in the pathogenesis of nephronophthisis. For example, *NPHP1* mutation causes nephronophthisis, whereas *NPHP6 *mutation alone does not lead to disease. In contrast, a combination of the same mutations in *NPHP1 *and *NPHP6* causes an additional extrarenal disease phenotype [89]. Other RDGs that interact genetically and may influence renal phenotype include *Pax2* and *LMX1B* or *Six1* and *Tbx18* [69, 70].

### 4.3. Epigenetic Modifiers

Great importance has been recently attributed to the epigenetic regulation of gene expression (epigenetic programming) and disease causality. The major mechanisms in epigenetic control of gene regulation are DNA or chromatin protein methylation and acetylation. Chromatin methylation and acetylation recruit additional proteins that can modify histones to form compact, inactive (heterochromatin), or opened, active (euchromatin), chromatin and alter RDG transcription [90, 91]. Specific combinations of these epigenetic marks determine whether to maintain a given RDG in an uncommitted transcriptional state with its transcripts present at low levels (poised state), stimulate its transcription by making it accessible to the transcription machinery, or silence it by packing into heterochromatin inaccessible to the transcription machinery [92]. Recent studies demonstrate that Pax2, a transcription factor critical for normal kidney development, is an important determinant of epigenetic marks during metanephric organogenesis [75]. Treatment of embryonic kidneys with inhibitors of histone deacetylases (HDACs), an evolutionary conserved group of enzymes that remove acetyl groups from histone tails, impairs UB branching and causes growth arrest and apoptosis [93]. Moreover, epigenetic programming may be inherited and may be involved in predisposition to complex diseases [94].

### 4.4. Mode of Inheritance

The mode of inheritance dictates the degree of genetic causality. In monogenic (Mendelian) recessive diseases, mutation in a given gene conveys a high risk of developing the disease by a defined age in early childhood. For example, in ARPKD disease-causing mutation conveys almost 100% risk of developing the disease [77, 79]. These diseases usually manifest complete penetrance (all individuals who have the disease-causing mutation have clinical symptoms of the disease) and present earlier in life. The strength of genotype-phenotype correlation is reduced in autosomal dominant, compared with recessive, diseases [77]. This may be due to incomplete penetrance or variable expression. This is also true for nonsyndromic forms of CAKUT such as unilateral and bilateral renal agenesis or severe dysplasia, most of which manifest autosomal dominant trait with penetrance between 50 and 90% and variable expression [95]. Genotype-phenotype correlations are the weakest in polygenic (complex) diseases, where mutations in multiple genes act in concert with environmental effects to cause a phenotype later in life. Although polygenic causation cannot be excluded in congenital solitary kidney, it is less likely since risks to offsprings are higher than expected for a strict multifactorial condition [30]. On the other hand, mutations in RDGs such as *Six2* and *Bmp4* are identified only in 3% of children from European population with nonsyndromic CAKUT that include unilateral renal agenesis and renal hypodysplasia [48]. High variability and low penetrance of *Six2* and *Bmp4 *mutations observed in this study are in accordance with the presumed polygenic inheritance of CAKUT. Unfortunately, such terms as “incomplete penetrance” or “variable expression” do not explain a biological mechanism but rather are labels for our ignorance.

### 4.5. Environment

**Figure 1:**
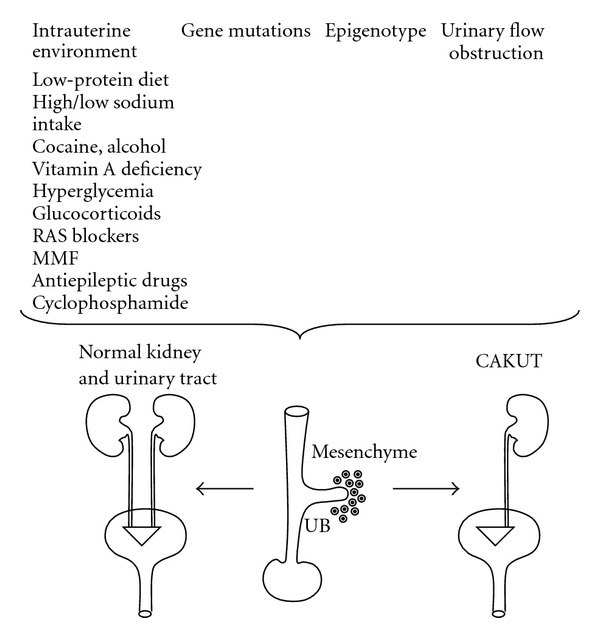
Schematic representation of the proposed impact of intrauterine environment, gene mutations, epigenotype, and urinary flow obstruction on the pathogenesis of CAKUT. These factors cause aberrant interactions among the mesenchyme, ureteric bud (UB), or bladder anlagen to result in CAKUT [102]. Please see text for details.

Intrauterine environment has been linked to CAKUT. Maternal low-protein diet initiated at onset of pregnancy in mice alters expression of RDGs in the embryonic metanephros and reduces nephron number [76]. One mechanism by which maternal low-protein diet may cause renal hypoplasia is by increasing concentration of glucocorticoids *via* downregulation of placental steroid-metabolizing enzyme 11 *β* -hydroxysteroid dehydrogenase type 2 [96]. Another mechanism may involve downregulation of angiotensin II contents in the embryonic kidney [97]. Both excessively high and low maternal sodium intake during pregnancy in the rat cause aberrant expression of critical RDGs and reduce the final number of glomeruli in the offspring, predisposing to hypertension later in life [98]. Additional factors that have been shown to result in CAKUT in children include maternal use of cocaine or alcohol during gestation (Figure 1) [99, 100]. Occurrence of renal hypodysplasia caused by high maternal salt intake during gestation in bradykinin *B2 receptor*-deficient mice provides proof of the principle that environmental factors may act in concert with single-gene mutations to cause CAKUT [101]. The mechanistic basis for CAKUT associated with altered intrauterine environment remains to be elucidated further.

## 5. Diagnostic Genomics Technologies in CAKUT

Three novel techniques are now available to accelerate discovery of causative genes in nonsyndromic CAKUT: genome-wide association studies (GWASs), exome capture, and next-generation DNA sequencing. GWASs avoid candidate-gene approach and map whole genomic DNA with markers to find loci (most commonly by genotyping single-nucleotide polymorphisms (SNPs)) associated with or in linkage disequilibrium (occurrence of some combinations of alleles or genetic markers in a population more often or less often than would be expected from a random formation of haplotypes from alleles based on their frequencies) with CAKUT. Although the ability of GWASs to identify the impact of common and rare variants on nonsyndromic CAKUT remains to be determined, GWASs generally rarely succeed in securely implicating specific genes in specific polygenic (common) diseases [103]. Exome capture and next-generation sequencing represent the most comprehensive study of the role of genetic variations in disease. Exome represents protein-coding subset of a genome. Because exons harbor 85% of mutations in single-gene diseases [104], exome capture (DNA hybridization with human exome array followed by amplification of captured DNA fragments) with consecutive next-generation sequencing (massively parallelized sequencing of captured and amplified DNA fragments) will help to identify CAKUT-causing alleles [105]. Although these techniques exemplify a fundamental advance for nephrology research, they are costly and require specific bioinformatic software for stringent data analysis, interpretation and reporting, and a large number of patients to yield adequate statistical power.

## 6. Implications of the State of Current Knowledge Regarding Genetic Cause of CAKUT

The cause of most cases of nonsyndromic CAKUT remains unknown. These types of CAKUT are assumed to be multifactorial and occur as a result of combination of epigenetic and environmental factors affecting genetically susceptible individuals. It is conceivable that polymorphism in a single given RDG may be in linkage disequilibrium with a separate, causative, mutation in a nearby gene. Perhaps polymorphisms or mutations in other genes must coexist to result in CAKUT. Application of GWASs, exome (and subsequently whole genome) capture and next-generation sequencing studies using the proper curation of CAKUT phenotypes, a family-based research design and properly-powered patient sample size will assist in identification of specific genetic determinants underlying nonsyndromic CAKUT and assess their causality. Establishment of collaborative framework among multiple centers throughout the world is required to unravel the genetic basis of CAKUT and provide precise genetic counseling for CAKUT patients and their relatives to enable personalized medical care based on the detailed understanding of the molecular pathogenesis of the disease.

## Figures and Tables

**Table 1:** Prevalence of CAKUT.

Type of malformation	Prevalence	References
Unilateral renal agenesis		
* Fetuses*	0.008%	[8]
* Newborns*		[11]
Bilateral renal agenesis		
* Fetuses*	0.013%	[8]
* Newborns*	1 in 30,000	[11]
Congenital hydronephrosis	1 in 1,000 live births	[12]
Renal hypodysplasia		
* Fetuses*	0.027%	[8]
* Newborns*	1 in 400 live births	[8]
Horseshoe kidney	1 in 1,000 newborns	[13]
PUV		
* Fetuses*	0.003%	[8]
VUR		
* Infants with hydronephrosis on* * prenatal ultrasonography*	3–19%	[14]
* Well children*	1–2%	
* Children with UTI*	25–40%	
Unilateral duplex ureter	1–8%	[15]

**Table 2:** Single-gene mutations associated with nonsyndromic human CAKUT.

Gene	Disease OMIM	Chromosome	Renal phenotype	Extrarenal phenotype	References
*AGT*	RTD	1p42	Reduced number of proximal tubules, short proximal tubules without brush border, atrophic loops of Henle and collecting ducts, closely packed glomeruli, marked thickening and disorganization of interlobular and preglomerular arteries	Large low-set ears, limb-positioning defects, arthrogryposis, lung hypoplasia, skull ossification defects	[29, 45]
*AGTR1*	RTD	3p24	Similar to *AGT *phenotype, PUV	Similar to *AGT* phenotype	[29, 45, 46]
*AGTR2*	—	Xq22-q23	UPJ obstruction, megaureter, MCDK hydronephrosis, PUV	—	[38–41, 47]
*ACE*	RTD	17q23.3	Similar to AGT phenotype renal hypodysplasia, PUV	Similar to AGT phenotype	[29, 45, 46]
*BMP4*	—	14q22-q23	Renal hypodysplasia	Cleft lip, microphthalmia	[48]
					
*BMP7* *Dlx5/Dlx6* *p63*	SHFM #603273	3q27	Urethral malformations	Split-hand/split-foot malformation	[49]
*CDC5L*	—	46XX,t(6; 19) (p21; q13.1)	Multicystic kidney dysplasia	—	[50]
*Eya1*	BOR #113650	8q12	Unilateral or bilateral renal agenesis renal hypodysplasia, VUR	Deafness, ear malformations branchial cysts	[51]
*Fras1/* *Fram2*	Fraser syndrome	4q21 13q13. 3	Renal agenesis/hypodysplasia	Ear and heart defects, syndactyly cryptophthalmos	[21, 51, 52]
*FoxC1*	—	6p25	CAKUT	—	[53]
*Gata3*	HDR syndrome #146255	10pter	Renal dysplasia	Hypoparathyroidism sensorineural deafness	[54]
*HNF1*β*/* *TCF2*	MODY5 #606391 RCAD #137920 GCKD #609886	17q12	Renal hypodysplasia, cysts	Diabetes	[20, 55, 56]
*Pax2*	Renal-coloboma syndrome	10q24	Renal hypoplasia, VUR	Optic nerve coloboma branchyal cysts	[34, 57, 58]
*Ren*	RTD	17q23.3	Similar to *AGT* phenotype	Similar to *AGT* phenotype	[29, 45]
*Ret*	Renal agenesis #191830	10q11.2	Absence of the kidney and ureter	Hirschsprung disease	[37]
*Robo2*	—	3p12.3	VUR	Limb and facial defects	[44, 59–61]
*Six2*	—	2p16-p15	Renal hypodysplasia	—	[48]
*Slit2*	—	4p15.2	Hydroureter, supernumerary UBs	—	[59]
*Umod*	MCDK2	16p12.3	Cysts in distal tubules and collecting ducts, renal dysplasia	—	[62, 63]
*Upk3A*	—	22q13.31	Renal agenesis/hypodysplasia	Facial and limb defects	[64, 65]
*Usf2*	—	46XX t(6;19) (p21; q13.1)	Multicystic kidney dysplasia	—	[66]
*XPNPEP3*	NPHP-like nephropathy	22q13.2	Renal cysts and dysplasia	—	[67]

AGTR: angiotensin II receptor type 1, AGTR2: angiotensin II receptor type 2, ARPKD: autosomal-recessive polycystic kidney disease, ADPKD: autosomal-dominant polycystic kidney disease, UPJ: ureteropelvic junction, VUR: vesicoureteral reflux, PUV: posterior urethral valves, UPJ: ureteropelvic junction, MCDK: multicystic dysplastic kidney, PUV: posterior urethral valve, RTD: renal tubular dysgenesis, RCAD: renal cysts and diabetes, MODY: maturity-onset diabetes, GCKD: glomerulocystic kidney disease, and NPHP: nephronophthisis, X-prolyl aminopeptidase (aminopeptidase P) 3, putative.
